# Voluntary Wheel Running Does Not Alter Mortality to or Immunogenicity of Vaccinia Virus in Mice: A Pilot Study

**DOI:** 10.3389/fphys.2017.01123

**Published:** 2018-01-05

**Authors:** Brandt D. Pence, Melissa R. Ryerson, Ariana G. Bravo Cruz, Jeffrey A. Woods, Joanna L. Shisler

**Affiliations:** ^1^School of Health Studies, University of Memphis, Memphis, TN, United States; ^2^Center for Nutraceutical and Dietary Supplement Research, University of Memphis, Memphis, TN, United States; ^3^Department of Kinesiology and Community Health, University of Illinois Urbana-Champaign, Urbana, IL, United States; ^4^Integrative Immunology and Behavior Program, University of Illinois Urbana-Champaign, Urbana, IL, United States; ^5^Department of Microbiology, University of Illinois Urbana-Champaign, Urbana, IL, United States

**Keywords:** exercise, voluntary wheel running, infection, virus, vaccinia virus, mortality

## Abstract

Exercise has been shown to improve immune responses to viral infections and vaccines in several mouse models. However, previous pathogen studies have primarily used infections limited to the respiratory tract. Additionally, previous studies have utilized forced treadmill exercise paradigms, and voluntary wheel running (VWR) has been shown to have differential effects on the immune system in non-infection models. We examined whether VWR could improve morbidity and mortality to a 50% lethal dose of vaccinia virus (VACV), a systemic pathogen commonly used to examine immune responses. Additionally, we examined whether VWR could improve antibody response to a replication-deficient strain of VACV, mimicking a vaccination. Male C57Bl/6J mice underwent 8 weeks of VWR or remained sedentary, then were infected intranasally with 10^5^ PFU VACV strain WR and followed 14 days for weight loss. Mice in the vaccination study ran or were sedentary for 8 weeks, then were given 10^6^ PFU of replication-deficient VACV strain MVA intraperitoneally. Blood was collected at 1, 2, and 4 weeks post-inoculation, and anti-VACV IgG titer was determined by ELISA. VWR did not improve mortality due to VACV infection (*p* = 0.26), although fewer VWR mice (4/10) died compared to sedentary (SED, 6/10). VWR did not prevent body weight loss due to infection compared to SED (*p* = 0.20), although VWR mice loss slightly less weight compared to SED through the first 6 days post-infection. Food intake was significantly reduced in SED post-infection compared to VWR (*p* = 0.05). VWR mice developed a greater IgG antibody response, although this was not significant (*p* = 0.22). In summary, VWR did not protect against mortality to VACV or prevent infection-induced weight loss, and VWR did not enhance antibody responses. However, there were non-significant trends toward VWR-related improvements in these outcomes, and post-infection food intake was improved by VWR.

## Introduction

Interventions which improve immune function and/or vaccine response, including exercise, are of critical importance in the field of public health. This is particularly true in populations at risk of poor immune responses, including individuals with obesity and individuals undergoing normal aging (Milner and Beck, 2012; Montecino-Rodriguez et al., 2013). However, prior to research in at-risk populations, the safety and efficacy of the combination of viral infection and exercise must be studied in healthy populations. These types of studies have been performed previously using influenza virus (Lowder et al., 2005, 2006; Sim et al., 2009) and herpes simplex virus (HSV)-1 (Kohut et al., 2001).

However, both influenza virus and HSV-1 are infections limited to the airway in mice. To date, no studies have been performed using systemic infections. Vaccinia virus (VACV) is a poxvirus which can be administered by a variety of routes and causes a systemic infection in mice (Hutchens et al., 2008). Poxviruses such as VACV are highly important from a public health perspective for several reasons. Historically, smallpox (variola virus) is potentially the most devastating human disease, with mortality rates of up to 30% prior to its eradication in 1980 (Nafziger, 2005). Despite the eradication of smallpox, there still exists a threat of the emergence of the disease or related poxviruses such as monkeypox, due either to natural mutations (in the case of monkeypox) or due to intentional release as biological weapons. Should such instances occur, vaccination with less-deadly poxviruses such as VACV is likely to be re-implemented.

Additionally, poxviruses such as wild-type and mutated VACV strains are commonly being used as vectors for vaccines for other pathogens (Lousberg et al., 2011) as well as for non-infectious diseases such as cancer (Kim and Gulley, 2012). VACV is highly genetically tractable, a major advantage in molecular biology research. VACV is a double-stranded DNA virus which encodes approximately 250 proteins, many with functions which modulate the host's immune response directly (Lousberg et al., 2011).

We undertook a small pilot study to examine the impact of exercise training on the immune response to VACV, using both a live viral infection model and an attenuated vaccine strain model in mice. Our goals were to establish whether exercise training was efficacious at improving immune responses to VACV. A secondary goal was to determine if moderate exercise was safe in the event of VACV exposure by infection or deliberate inoculation. We hypothesized that exercise training in mice would reduce viral infection-mediated mortality and improve the antibody response to the vaccine strain. A thorough understanding of the relationship between exercise training and the immune response to vaccinia virus could be important in the future both for understanding the basic mechanisms by which exercise alters immune function, as well as for ensuring the safety and efficacy of vaccinations with VACV-derived viral strains in the event they are reintroduced.

## Methods

### Mice

Six week old male C57Bl/6J mice (*N* = 38) were acquired from Jackson Laboratories (Bar Harbor, ME) and acclimated for 1 week in our facility prior to onset of the exercise protocol. All mice received *ad libitum* access to a rodent chow diet and water during the course of the study. Mice were housed on a 12-h light-dark cycle with lights on at 0800 each day. All experimental procedures were approved by the Institutional Animal Care and Use Committee at the University of Illinois Urbana-Champaign, and carried out in an AAALAC-accredited facility under biosafety level 2 conditions.

### Exercise

Mice exercised or remained sedentary for 8 weeks prior to VACV infection. The wheel running group (**Wh**) was given *ad libitum* access to stainless steel running wheels both for the 8 weeks prior to inoculation and for the period between inoculation and mortality/euthanasia. Sedentary mice (**Sed**) remained in their home cages for the duration of the study. All mice underwent similar handling.

### Viruses

Mice were infected with VACV strain Western Reserve (WR) for determination of morbidity and mortality responses. WR was cultured in BHK-1 cells (ATCC, Manassas, VA), titrated by plaque assay using standard techniques, and purified by ultracentrifugation. Mice were inoculated under anesthesia by inhaled isoflurane with 10^5^ PFU WR in 20 μl PBS, given intranasally as 10 μl per nostril. This dose of WR was verified to cause approximately 50% mortality in sedentary mice in a preliminary experiment prior to the study (data not shown). For antibody responses, mice were inoculated by intraperitoneal injection with 10^6^ PFU of replication-deficient Modified Vaccinia Ankara (MVA) virus in 100 μl PBS. MVA was cultured in CEF cells (ATCC), titrated by plaque assay, and purified by ultracentrifugation prior to inoculation.

### Morbidity and mortality

Morbidity was measured by examining reductions in daily food intake and body weights for 6 days post-infection with WR, as significant mortality took effect beginning on day 7 post-infection. Body weight was monitored by daily weighing with a digital scale. Food intake was measured by food disappearance by subtracting the current day's food weight from the previous day's food weight. Typical subjective scoring measures used to assess morbidity could not be used in this study due to the need to manipulate mice only under a biosafety cabinet, which precluded blinding of investigators to the presence or absence of a running wheel. For mortality, mice were euthanized once they reached a weight loss of 30% of pre-infection weight, the point at which WR-infected mice have been shown to fail to recover from viral infection (Bartlett et al., 2002). A small number of mice died naturally from WR infection prior to reaching this body weight cutoff.

### Antibody responses

Prior to MVA injection and at 1, 2, and 4 weeks post-inoculation, mice were anesthetized by inhalation with isoflurane in a flow rate of 2–3 L·min^−1^ oxygen. Blood (200 μl) was collected from the retro-orbital sinus with a glass Pasteur pipette into heparinized microcentrifuge tubes, then centrifuged at 1,500 × *g* for 15 min to separate plasma. Plasma was stored at −80°C.

Anti-vaccinia IgG response was determined by enzyme-linked immunosorbant assay. Briefly, 96-well plates (Corning, Corning, NY) were coated with PBS containing 1 μg whole cell lysate total protein from MVA-infected CEF cells or mock-infected CEF cells (as non-specific binding controls) and allowed to attach overnight at 4°C. Binding sites were then blocked for 1 h with 1% bovine serum albumin at room temperature, followed by addition of plasma diluted 1:100 in PBS. After a 2 h incubation at room temperature, plates were washed and 50 μl of 1:800 rabbit anti-mouse IgG-HRP (Life Techonologies, Frederick, MD) was added to each well. Plates were incubated for 1 h at room temperature and washed, followed by incubation for 20 min in 50 μl of TMB substrate reagent (1:1 mixture of TMB and hydrogen peroxide, BD Biosciences, San Jose, CA) and read at 405 nm on a spectrophotometric plate reader (BioTek, Winooski, VT).

Plasma IgG optical density (O.D.) from binding to mock infected CEF lysates was subtracted from that to MVA-infected CEF lysates to correct for non-specific antibody binding. Plasma IgG values were expressed as change in O.D. from the pre-inoculation measure.

### Data analysis

Data were analyzed with GraphPad Prism 5 (La Jolla, CA) unless otherwise noted. Mortality was assessed by the Mantel-Cox test, comparing survival portions of Wh vs. Sed. Body weight and food intake data during infection with WR were analyzed by repeated measures analysis of variance (RM-ANOVA), with Bonferroni *post-hoc* correction in the event of a significant main effect or interaction. Antibody response data were also assessed with RM-ANOVA using the same methods. For proportional tests for threshold antibody responses at week 4 post-inoculation, a Pearson's chi-square test was performed using R v. 3.2.1 (R Foundation, Vienna, AUS). For post-hoc power analyses using mortality and antibody response results, power was calculated using the “pwr” package in R v. 3.2.1. Significance was set at *p* ≤ 0.05.

## Results

### Morbidity and mortality

**Figure 1:**
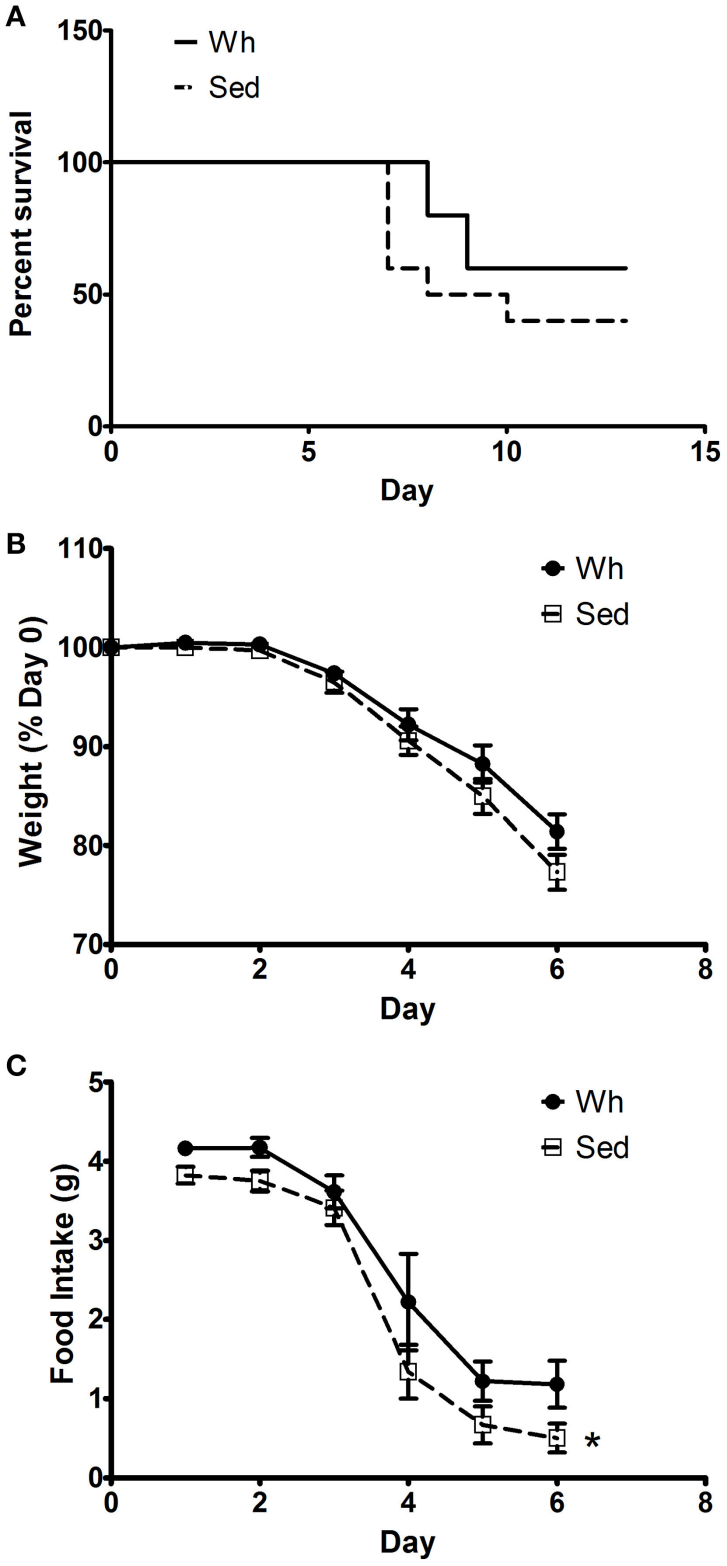
Morbidity and mortality response to infection with VACV strain WR. **(A)** Percentage survival by day post-infection. **(B)** Body weight as a percentage of pre-infection weight. **(C)** Daily food intake post-infection. *Main effect of activity (*p* < 0.05). Wh, wheel mice. Sed, sedentary mice. *N* = 10/group.

No significant interactions were recorded for morbidity and mortality metrics, therefore we report main effects of exercise in this section. Wh did not significantly improve mortality rates due to infection with WR, although mice in the Wh group did survive at a higher rate (60%) than those in the Sed group (40%, *p* = 0.26, Figure 1A). Additionally, body weight did not differ during infection with WR, although again the Sed group lost more 4% weight than those in the Wh group (*p* = 0.20, Figure 1B). Promisingly, food intake was significantly reduced in the Sed group compared to the Wh group (*p* = 0.05, Figure 1C), suggestive of a Wh effect on morbidity.

### Antibody responses

**Figure 2:**
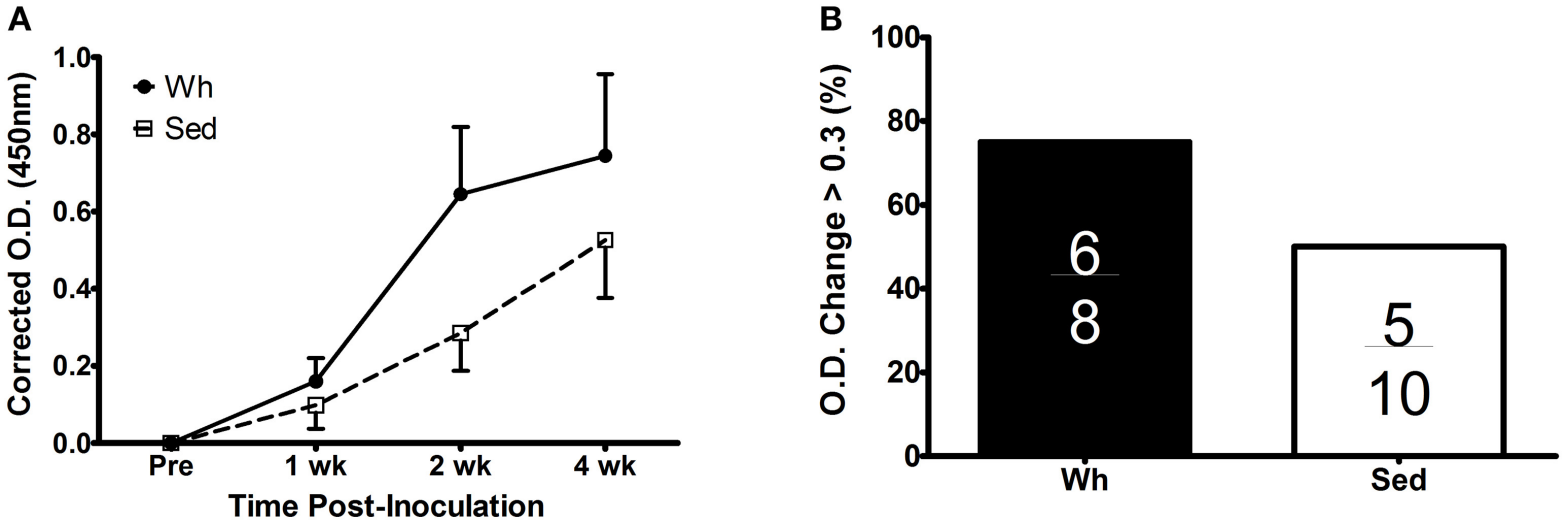
Antibody response to inoculation with VACV strain MVA. **(A)** Plasma anti-VACV IgG at pre-inoculation and at 1, 2, and 4 weeks post-inoculation, corrected for non-specific binding and expressed as difference from baseline **(B)** Proportion of mice with O.D. increase of >0.3 from pre-inoculation to 4 weeks post-inoculation. Inset fractions indicate mice meeting the change criteria out of total mice in the group. Wh, wheel mice (*N* = 8). Sed, sedentary mice (*N* = 10). O.D., optical density.

We examined the kinetic antibody response to intraperitoneal injection of MVA by ELISA (Figure 2A). Inoculation of MVA induced a significant increase in anti-VACV IgG in both groups (overall time main effect *p* < 0.001). Although the Wh group had greater induction of IgG response, especially at later time points (i.e., week 2 and week 4 post inoculation), the main effect of exercise was not significant (*p* = 0.22), likely due to the high variability of antibody response. We also examined the proportion of responses in which O.D. increased by >0.3 from pre-inoculation to week 4 post-inoculation (Figure 2B). Although 6/8 Wh mice met this standard, compared to only 5/10 Sed mice, this proportion was not significantly different by Pearson's chi-squared test (*p* = 0.28).

## Discussion

Exercise has long been known to have immunomodulatory properties which alter the response of the host to viral infections. Early work by Nieman and colleagues demonstrated that higher levels of exercise training reduced symptoms of upper respiratory tract infection (Nieman et al., 1989), and that exercise training interventions could similarly reduce the rate of reported symptoms (Nieman et al., 1990, 1993). Because human studies are generally limited to symptom reports for ethical reasons, and because reported symptom are not necessarily related to actual pathogen exposure (Spence et al., 2007), experimental studies using viral infections in rodent models were undertaken by several investigators in order to directly study the effects of exercise on viral infections.

One of the earliest studies along these lines (Kohut et al., 2001) demonstrated that 8 weeks of moderate treadmill training enhanced antiviral antibody responses and production of Th1-associated cytokines in aged mice in response to HSV-1. Additionally, a four-day moderate exercise program (30 min·day^−1^) given post-infection (but prior to symptom onset) reduced mortality to influenza virus in mice (Lowder et al., 2005), while a 3.5 month chronic exercise training program showed similar benefits in mice exposed to influenza virus infection (Bartlett et al., 2002).

The above studies share several characteristics, most notably that (i) exercise was conducted using forced treadmill running, and (ii) viral infections were limited to the respiratory tract. To date, we are aware of no research which has examined the impact of VWR on the response to viral infection in mice. This is important, as previous research has demonstrated that VWR and forced treadmill running can modulate the immune response in different (and indeed opposite) ways (Cook et al., 2013). Therefore, we chose to use VWR as our exercise model for this pilot study. Additionally, we chose to use VACV, a virus that is not limited to the respiratory tract, as a more general model for viral infections in this study.

In short, we found that VWR in mice did not alter the antibody response to the attenuated MVA strain of VACV, nor did it change the morbidity or mortality response to the pathogenic WR strain. However, we noted general trends for improvements with exercise, both in reducing morbidity and mortality to WR and in enhancing anti-VACV IgG responses.

It appears that the variability in the responses in these mice makes it difficult to confirm differences in small sample sizes, such as the 8-10 per group used in this study. Sensitivity analyses suggest that 60 mice per group would be necessary to conclude that exercise is protective against mortality from WR infection, and that more than 120 mice per group would be necessary to conclude that antibody responses are different at 4 weeks post-MVA inoculation. A study of this magnitude is impractical and would have low benefit-to-cost ratio, given the variability and small effect sizes seen here. Effectively, the use of so many additional mice to support what is likely a small (and potentially not physiologically-relevant) exercise effect in this case is, in our view, unethical in the light of significant burden to the additional mice for little gain.

It is likely that young healthy mice, such as those used in this study, have sufficiently strong immune responses such that exercise is not enough to induce a large improvement. This exercise paradigm may be more efficacious in models with impaired immune responses to viral infections, such as aging (Montecino-Rodriguez et al., 2013) or obesity (Milner and Beck, 2012). Exercise has recently been shown to enhance immunity to influenza infection in obese mice (Warren et al., 2015), and a similar effect is possible using this VACV infection model.

Despite the non-significant findings in this study, our results do demonstrate relatively convincingly that moderate exercise training is unlikely to either decrease the safety and efficacy of a VACV-based vaccination or to enhance the pathogenicity of a poxviral infection. These outcomes are important, given the increasing use of MVA and similar attenuated VACV strains in experimental vaccines (Hutchens et al., 2008; Lousberg et al., 2011).

In conclusion, moderate exercise training by voluntary wheel running did not alter morbidity or mortality to infection with pathogenic VACV strain WR, and did not alter antibody responses to a vaccination challenge with attenuated VACV strain MVA. Small effect sizes and high variability obscured the general trend toward improved responses with exercise in this study, and future research in models with impaired immune responses may hold more promise.

## Author contributions

BP conceived the study. BP, JW, and JS designed the study. BP, MR, and AB collected data. BP analyzed and interpreted the data and drafted the manuscript. BP, MR, AB, JW, and JS revised the manuscript and approved the final version.

## Conflict of interest statement

The authors declare that the research was conducted in the absence of any commercial or financial relationships that could be construed as a potential conflict of interest.
